# Green biofuels and bioproducts: bases for sustainability analysis

**DOI:** 10.1111/1751-7915.12768

**Published:** 2017-07-17

**Authors:** Juan L. Ramos, Francisco García‐Lorente, Miguel Valdivia, Estrella Duque

**Affiliations:** ^1^ Department of Biotechnology Abengoa Research Avenida de la Energía Solar, 1. Building D 41014 Sevilla Spain

## Abstract

Currently the chemical industry is largely petroleum based and although the number of ongoing large‐scale biocatalytic processes are relatively low, a trend in growth is expected and the Organization for Economic Co‐operation and Development (OECD) and other agencies aim to have 30% of the total chemical industry based on renewable sources by 2050 (Philp *et al*., 2013). At present a good number of bio‐based products (bioethanol, acids such as lactic, succinic, itaconic and others) are derived from corn syrup and other sugar sources (Geiser *et al*., 2016; Ramos *et al*., 2016a); however, because of the food v fuel controversy new trends have been directed towards the production of bioproducts/biofuels from lignocellulosic biomass—the most abundant and important renewable source for alternative petrol derivatives. We discuss here the bases for sustainable bioenergy production.

Currently, the chemical industry is largely petroleum based and although the number of ongoing large‐scale biocatalytic processes is relatively low, a trend in growth is expected and the Organization for Economic Co‐operation and Development (OECD) and other agencies aim to have 30% of the total chemical industry based on renewable sources by 2050 (Philp *et al*., 2013). At present, a good number of bio‐based products (bioethanol, acids such as lactic, succinic, itaconic, and others) are derived from corn syrup and other sugar sources (Geiser *et al*., 2016; Ramos *et al*., 2016a); however, because of the food v fuel controversy, new trends have been directed towards the production of bioproducts/biofuels from lignocellulosic biomass – the most abundant and important renewable source for alternative petrol derivatives (Ramos *et al*., 2016b). Lignocellulosic biomass is the stored energy from sunlight in the form of chemical energy, and it refers to plant biomass that is composed of cellulose, hemicellulose and lignin and includes not only the green parts of vegetables, wood and straw, but also manure and the organic fraction of municipal solid wastes (MSW). The term biofuel includes liquid biofuels; i.e. starch‐based and cellulose‐based ethanol, biogas, renewable thermal energy, renewable electricity and bio‐based adhesives, among others (Arjona‐Antolin *et al*., 2012a,b,c,d). In turn, the term bioproduct refers to bioplastics, a range of acids (citric, lactic), surfactants resins and biochemicals. In Europe, there is a slight bias towards the development of bioproducts, and in North America, a clear tendency to produce biofuels.

A major obstacle for the industrial‐scale production of bioproducts/biofuels from lignocellulose by biological means, known as second‐generation (2G) products, is the inefficient deconstruction of plant material due to the recalcitrant nature of the substrates (Horn *et al*., 2012; Cherry and Fidantsef, 2013; Alvarez *et al*., 2016). The success of 2G technology requires efficient pre‐treatment (physical, chemical or physicochemical) of plant material to disorganize the fibres, which will be the target for the action of enzymatic cocktails that breakdown polysaccharides into their monomeric constituents (Alvarez *et al*., 2016). Enzymatic hydrolysis of lignocellulosic materials yields glucose, xylose and arabinose, and these sugars can be fermented to produce added‐value chemicals such as alcohols (ethanol, butanol), acetone, aldehydes, amino acids and other bioproducts.

Liquid or gas bioproducts/biofuels, derived from renewable resources, can only replace a fraction of the fossil fuels used in locomotion, as well as a number of chemicals that are currently derived from petroleum. In the field of bioproduct/biofuel production from renewable plant material, the most relevant advances have been reported for 2G bioethanol production (Gnansounun and Dauriat, 2010), for which, globally, there are two commercial plants in operation.

Biofuels are gaining increased public acceptance and scientific attention, driven by factors such as oil price spikes, the need for increased energy security and concerns over greenhouse gas (GHG) emissions from fossil fuels (Valdivia *et al*., 2016). In addition to the lower GHG emissions from using bioproduct/biofuel, petroleum saved for locomotion will allow this valuable resource to be used as ‘reserve’ for the future. Biofuels are used among others for ethyl tert‐butyl ether (ETBE) production (gasoline additive), direct blending of ethanol with gasoline or blending of biodiesel with diesel. Being a renewable energy source, biofuels reduce CO_2_ emissions and contribute to the security and diversification of the energy supply, while reducing the dependency on fossil fuels and helping towards compliance with international Climate Agreements and Protocols. Bioproducts are also gaining interest, and a number chemicals are being synthesized by direct fermentation of 2G sugars, i.e. butanol, acetone, ethanol from corn stover (Wang and Chen, 2011; Geiser *et al*., 2016).

The biomass that is used for biofuel/bioproduct synthesis should be of sustainable origin and deemed to comply with the established sustainability requirements for biodiversity, carbon stock, peatland and land‐use change (Arjona‐Antolin *et al*., 2012a,b,c,d). A system to identify sustainable geographical areas has been developed by Abengoa (Spain) based on a photographic system adapted to capture images of the areas of interest furnished with an image transmission methodology for transmitting the captured images (Arjona‐Antolin *et al*., 2012a,d). The system uses historical databases of the geographical areas, the strategy followed for the identification of sustainable geographical areas comprises: (i) capturing satellite images for the studied region, in medium/high resolution, and the analysis of historical archives of images available in official databases; (ii) land‐use analysis, which uses satellite images imported into an image processing module, and pre‐processing of the images to obtain a land‐use classification using the parameters defined by sustainability requirements in one map, and the six categories used by IPCC plus a seventh category of perennial crops in another map (Lapola *et al*., 2010). The image classification system also defines the land cover for each area. Following processing and determination of matching/non‐matching land use, the results are displayed (Arjona‐Antolin *et al*., 2012b). The output provides critical information that allows one to demonstrate fulfilment of the land‐use requirements.

The primary greenhouse gases in the Earth`s atmosphere are carbon dioxide, methane and nitrous oxide; the greenhouse gas (GHG) emissions released during biofuel/bioproduct production from different source materials must be determined in order to complete sustainability analysis calculations (Arjona‐Antolin *et al*., 2012a,c). The biofuel/bioproduct generation sustainability analysis also needs to consider the source material and the separation of target products and co‐products. For each of the potential steps, the energy and raw material consumption as well as the wastes generated have to be identified and calculated. Another relevant issue is the consideration of the GHG emissions related to transportation and logistics required to obtain the source material and transport the bioproduct following processing.

The method developed by Abengoa to measure the sustainability of biofuels/bioproducts comprises capturing, processing and handling different parameters and data related to the GHG emissions associated with every single step in the process needed to produce a given bioproduct/biofuel. The method estimates a total GHG emission value by means of applying formulae to the processed isolated GHG emission data from each of the steps in the process. GHG emissions associated with the entire biofuel/bioproduct process are calculated according to the following formula, where ‘*n*’ represents previously identified steps:

$${\text{Emission}}_{\mathrm{Taski}}=\sum_{i=1}^{n}\left({\mathrm{ActivityData}}_{i}.{\mathrm{EmissionFactor}}_{i}\right)$$

in which:

Activity data is a characteristic parameter of the activity or tasks of the equipment, installations, processes or vehicles associated with a given source, which through calculation allows determination of their emissions for a given period. Examples of activity data are the fuel consumption, the consumption of raw materials and the distance covered by vehicles. The value of each activity data could vary in each production plant and depending on the raw material type used in the process.

The Emission Factor is a parameter that indicates the quantity of a particular contaminant emitted from an activity per unit of product, volume, duration, quantity of raw material or fuel. The value of each emission factor may also vary in each production plant and according to the raw material used.

As indicated above in the process of biofuel/bioproduct production, a series of co‐products are often obtained. The emissions associated with them also have to be taken into account; in some cases, the co‐product may be pure waste, such as sewage water, but in others may be valuable products, such as, lignin cake, Distilled Dry Grains with Solubles (DDGS), which would need individual treatments to be sold, i.e. solid/liquid separation, evaporation, drying and pelletization; in both cases, emissions related to these are also accounted for in the sustainability calculations.

A current trend is the use of MSW for production of biofuels/bioproducts, and this is in agreement with the principles of the circular economy. The advantages that MSW has over other sources of organic material are as follows it is available throughout the year, it is concentrated (supply locations), and it is less costly or even a direct source of revenues for processing companies due to the cost paid for its disposal (Arena *et al*., 2015). In accordance with the European waste hierarchy, only the non‐recyclable fraction of MSW, called MSW refuse, can be directly used for energy recovery, including electricity and fuel production; while the use of other waste fractions must be justified by a life cycle assessment (LCA, European Parliament, 2008). LCA supports that the global GHG emission from converting the organic matter of wastes into ethanol is favourable compared with the GHG emissions to obtain the same ‘energy’ from fossil fuels, and this technology will be promoted in future as a source of liquid biofuels.

Aracil *et al*. ([Link]) assessed the potential climate benefit of biofuel production from wastes; their results revealed that in countries with negligible landfill, the production of biofuels would lead to a clear immediate climate benefit. For landfill‐dominant countries, the climate benefit would be achieved in the medium term as the impact of landfills on climate decreases over time. In Europe and the USA, a progressive banning of landfill has been set by policies for environmental protection and resource efficiency. Under these circumstances, the results will positively impact the climate change mitigation guaranteed by using MSW for biofuel production and will be particularly encouraging when fuel and ethanol/butanol or other chemicals are produced.

## Conflict of interest

None declared.
